# Supplementary material for: C-Type Natriuretic Peptide Acts as a Microorganism-Activated Regulator of the Skin Commensals Staphylococcus epidermidis and Cutibacterium acnes in Dual-Species Biofilms
Source: Biology (Basel). 2023 Mar 12;12(3):436. doi: 10.3390/biology12030436 (PMC10045295; doi:10.3390/biology12030436)
Supplement: Supplementary file 1 [file biology-12-00436-s001.zip › Supplementary Table 1.pdf]

Supplementary Table 1. Differential expression of genes in *S. epidermidis* under anaerobic conditions in comparison to samples cultivated aerobically

Locus tag	Protein description	log2 (expression level ratio)	Standard error of log2	p-value	q-value	Conclusion about expression difference
**B6C95_00465**	dihydrolipoyl dehydrogenase	2.38574	0.297895	3.29×10^{-6}	0.000246	higher
**B6C95_01415**	hypothetical protein	6.169337	1.655147	0.001789	0.04734	higher
**B6C95_02220**	hypothetical protein	3.447159	0.597661	4.23×10^{-5}	0.002079	higher
**B6C95_02455**	hypothetical protein	6.922889	1.807026	0.001047	0.031502	higher
**B6C95_02685**	PTS maltose transporter subunit IIBC	2.925258	0.456733	2.49×10^{-5}	0.001335	higher
**B6C95_03235**	hypothetical protein	5.561468	0.67508	1.41×10^{-11}	1.13×10^{-8}	higher
**B6C95_04650**	sodium:proton antiporter	2.084589	0.268427	5.33×10^{-5}	0.002532	higher
**B6C95_04715**	hypothetical protein	9.391025	2.14555	9.2×10^{-5}	0.003885	higher
**B6C95_04760**	hypothetical protein	9.69319	1.506041	7.82×10^{-9}	3.14×10^{-6}	higher
**B6C95_05315**	riboflavin synthase	3.702796	0.728696	0.000208	0.008294	higher
**B6C95_05325**	6.7-dimethyl-8-ribityllumazine synthase	3.526783	0.721946	0.000465	0.016007	higher
**B6C95_05760**	cytochrome C assembly protein	1.891652	0.283093	0.001635	0.044726	higher
**B6C95_06190**	hypothetical protein	7.901612	2.047588	0.00075	0.023733	higher
**B6C95_06475**	hypothetical protein	4.646343	1.164413	0.001739	0.047057	higher
**B6C95_06835**	hypothetical protein	8.365145	1.789596	3.86×10^{-5}	0.001938	higher
**B6C95_07015**	argininosuccinate lyase	4.002439	0.757641	7.41×10^{-5}	0.003302	higher
**B6C95_07020**	argininosuccinate synthase	4.8637	0.746392	2.26×10^{-7}	3.2×10^{-5}	higher
**B6C95_07125**	ABC transporter ATP-binding protein	4.237828	0.998466	0.001184	0.034123	higher
**B6C95_07655**	cytochrome ubiquinol oxidase subunit I	3.752818	0.647512	2.12×10^{-5}	0.001163	higher
**B6C95_07660**	cytochrome D ubiquinol oxidase subunit II	2.996862	0.608105	0.001024	0.031502	higher
**B6C95_07815**	acetolactate synthase	9.953579	2.234645	6.16×10^{-5}	0.002797	higher
**B6C95_07820**	acetolactate synthase. large subunit. biosynthetic type	4.781712	1.027791	0.000234	0.008934	higher
**B6C95_07965**	hypothetical protein	9.529666	1.798466	2.11×10^{-6}	0.000169	higher
**B6C95_07970**	GntR family transcriptional regulator	7.440111	1.737495	0.00021	0.008294	higher
**B6C95_07980**	hypothetical protein	13.03266	2.393732	4.99×10^{-7}	5.72×10^{-5}	higher

**B6C95_07985**	ABC transporter ATP-binding protein	7.31135	1.122844	1.9×10^{-8}	4.92×10^{-6}	higher
**B6C95_07990**	ABC transporter permease	7.413545	1.965368	0.001101	0.032341	higher
**B6C95_07995**	hypothetical protein	5.368595	1.358882	0.001305	0.036544	higher
**B6C95_08000**	hypothetical protein	6.13914	1.272311	5.36×10^{-5}	0.002532	higher
**B6C95_08005**	hypothetical protein	11.15127	2.223702	4.99×10^{-6}	0.000352	higher
**B6C95_08010**	hypothetical protein	12.78413	2.643768	8.3×10^{-6}	0.000555	higher
**B6C95_08020**	hypothetical protein	9.641623	1.975022	1.21×10^{-5}	0.000768	higher
**B6C95_08030**	transposase	7.96256	1.980567	0.000439	0.015322	higher
**B6C95_08035**	transposase	12.53222	2.216098	1.95×10^{-7}	3.2×10^{-5}	higher
**B6C95_08040**	transposase	13.12045	2.595513	3.02×10^{-6}	0.000234	higher
**B6C95_08045**	hypothetical protein	12.0911	2.142415	2.26×10^{-7}	3.2×10^{-5}	higher
**B6C95_08050**	hypothetical protein	14.3729	2.572703	2.01×10^{-7}	3.2×10^{-5}	higher
**B6C95_08060**	BlaR1 family beta-lactam sensor/signal transducer	9.068158	2.372682	0.000673	0.021601	higher
**B6C95_08065**	penicillinase repressor BlaI	9.163875	2.083319	8.9×10^{-5}	0.003828	higher
**B6C95_08380**	hypothetical protein	3.069057	0.665305	0.001871	0.048978	higher
**B6C95_08575**	multidrug resistance protein	9.677247	1.767412	9.13×10^{-7}	8.2×10^{-5}	higher
**B6C95_08580**	hypothetical protein	8.485508	1.726865	1.46×10^{-5}	0.000864	higher
**B6C95_08585**	multidrug resistance protein	8.940916	2.189809	0.000288	0.010651	higher
**B6C95_08590**	PTS-dependent dihydroxyacetone kinase phosphotransferase subunit DhaM	13.74619	2.122209	1.9×10^{-9}	9.15×10^{-7}	higher
**B6C95_08595**	dihydroxyacetone kinase subunit L	12.94192	1.981614	1.68×10^{-9}	9.15×10^{-7}	higher
**B6C95_08600**	dihydroxyacetone kinase subunit DhaK	12.77922	2.336364	4.61×10^{-7}	5.72×10^{-5}	higher
**B6C95_08605**	glycerol dehydrogenase	12.82231	2.403124	8.67×10^{-7}	8.2×10^{-5}	higher
**B6C95_08945**	formate dehydrogenase subunit delta	8.214078	1.991664	0.000292	0.01066	higher
**B6C95_09800**	hypothetical protein	3.429463	0.560693	1.47×10^{-5}	0.000864	higher
**B6C95_09835**	tRNA-Leu	4.337589	1.017876	0.001042	0.031502	higher
**B6C95_10320**	tRNA-Thr	7.689584	1.986591	0.000759	0.023733	higher
**B6C95_10555**	hypothetical protein	7.613918	1.914797	0.000552	0.018465	higher
**B6C95_10650**	hypothetical protein	8.827513	1.876613	3.03×10^{-5}	0.001587	higher

**B6C95_10660**	site-specific integrase	7.829247	2.10702	0.00119	0.034123	higher
**B6C95_11215**	tandem-type lipoprotein	9.700631	1.775297	9.54×10^{-7}	8.2×10^{-5}	higher
**B6C95_11225**	replication initiation protein	7.357231	1.295597	9.26×10^{-7}	8.2×10^{-5}	higher
**B6C95_11230**	universal stress protein	13.54555	2.700025	3.38×10^{-6}	0.000246	higher
**B6C95_11235**	type I restriction endonuclease	9.914077	1.787331	6.12×10^{-7}	6.7×10^{-5}	higher
**B6C95_11240**	hypothetical protein	9.689367	1.673333	2.07×10^{-7}	3.2×10^{-5}	higher
**B6C95_11245**	ArgR family transcriptional regulator	8.012969	2.024268	0.000531	0.01802	higher
**B6C95_11250**	arginine deiminase	13.54825	2.196236	1.11×10^{-8}	3.81×10^{-6}	higher
**B6C95_11255**	arginine-ornithine antiporter	13.90738	2.301473	2.04×10^{-8}	4.92×10^{-6}	higher
**B6C95_11260**	Crp/Fnr family transcriptional regulator	14.13672	2.589418	3.91×10^{-7}	5.23×10^{-5}	higher
**B6C95_11265**	ornithine carbamoyltransferase 1. catabolic	12.06961	2.293572	1.39×10^{-6}	0.000115	higher
**B6C95_11270**	carbamate kinase 2	12.97	2.427385	8.17×10^{-7}	8.2×10^{-5}	higher
**B6C95_11525**	hypothetical protein	7.641479	1.932517	0.000589	0.019424	higher
**B6C95_11755**	hypothetical protein	4.77807	0.705221	8.45×10^{-8}	1.7×10^{-5}	higher
**B6C95_11760**	hypothetical protein	3.064075	0.646882	0.001419	0.039269	higher
**B6C95_11965**	pyridine nucleotide-disulfide oxidoreductase	13.11977	2.154301	1.85×10^{-8}	4.92×10^{-6}	higher
**B6C95_11970**	Hg(II)-responsive transcriptional regulator	8.61243	1.931629	8.12×10^{-5}	0.003553	higher
**B6C95_11975**	hypothetical protein	10.47813	1.882378	4.77×10^{-7}	5.72×10^{-5}	higher
**B6C95_11980**	cytochrome C biosynthesis protein	11.8098	2.520387	1.8×10^{-5}	0.001029	higher
**B6C95_11985**	mercury transporter	9.468743	2.289402	0.000216	0.008403	higher
**B6C95_11990**	mercury(II) reductase	39.3922	2.868794	7.63×10^{-41}	1.84×10^{-37}	higher
**B6C95_11995**	alkylmercury lyase	38.78754	2.915048	1.99×10^{-38}	2.39×10^{-35}	higher
**B6C95_12000**	hypothetical protein	7.940991	1.889769	0.00024	0.009022	higher
**B6C95_12005**	hypothetical protein	6.903998	1.343231	1.11×10^{-5}	0.00072	higher
**B6C95_12010**	hypothetical protein	11.33396	1.861533	2.84×10^{-8}	6.21×10^{-6}	higher
**B6C95_12015**	hypothetical protein	11.64303	2.333939	5.11×10^{-6}	0.000352	higher
**B6C95_12040**	MurR/RpiR family transcriptional regulator	2.496946	0.344961	1.43×10^{-5}	0.000864	higher
**B6C95_12145**	hypothetical protein	8.671982	1.801372	2.05×10^{-5}	0.00115	higher
**B6C95_12150**	hypothetical protein	8.013015	1.859283	0.000162	0.006612	higher
**B6C95_12160**	IS5/IS1182 family transposase	3.311866	0.67389	0.000602	0.019595	higher

**B6C95_00780**	RNase P RNA component class B	−2.60343	0.495541	0.001213	0.034373	lower
**B6C95_02950**	amino acid ABC transporter permease	−3.35398	0.65438	0.000322	0.011558	lower
**B6C95_03530**	NifU family protein	−3.07063	0.515192	5.84×10^{-5}	0.002705	lower
**B6C95_04975**	3-hexulose-6-phosphate synthase	−2.88535	0.456413	3.62×10^{-5}	0.001852	lower
**B6C95_05945**	6S RNA	−3.68424	0.822082	0.001094	0.032341	lower
**B6C95_06070**	signal recognition particle sRNA large type	−4.69053	0.749379	8.45×10^{-7}	8.2×10^{-5}	lower
**B6C95_06795**	glycosyl transferase family 2	−2.20415	0.385214	0.001772	0.04734	lower
**B6C95_09070**	sugar dehydrogenase	−2.45317	0.380604	0.000135	0.005584	lower
**B6C95_12205**	16S ribosomal RNA	−5.65313	1.319899	0.000423	0.014975	lower
