# Supplementary material for: C-Type Natriuretic Peptide Acts as a Microorganism-Activated Regulator of the Skin Commensals Staphylococcus epidermidis and Cutibacterium acnes in Dual-Species Biofilms
Source: Biology (Basel). 2023 Mar 12;12(3):436. doi: 10.3390/biology12030436 (PMC10045295; doi:10.3390/biology12030436)
Supplement: Supplementary file 1 [file biology-12-00436-s001.zip › Supplementary Table 2.pdf]

Supplementary Table 2. Differential expression of genes in *C. acnes* in dual-species biofilms in comparison to *C. acnes* monospecies biofilms

Locus tag	Protein description	log2 (expression level ratio)	Standard error of log2	p−value	q−value	Conclusion about expression difference
**HMPREF9571_RS01520**	anchored repeat-type ABC transporter ATP-binding subunit	3.807799	0.654751	1.8×10^{-5}	0.001335	higher
**HMPREF9571_RS01525**	choice-10-of-anchor M domain-containing protein	4.643417	0.680429	8.58×10^{-8}	9.13×10^{-6}	higher
**HMPREF9571_RS01530**	anchored repeat ABC transporter. substrate-10-binding protein	5.55488	0.702117	8.74×10^{-11}	1.53×10^{-8}	higher
**HMPREF9571_RS01535**	TIGR03773 family transporter-associated surface protein	6.856548	0.564467	3.21×10^{-25}	7.86×10^{-22}	higher
**HMPREF9571_RS02495**	metal ABC transporter permease	4.591287	0.543158	3.8×10^{-11}	9.29×10^{-9}	higher
**HMPREF9571_RS02500**	ABC transporter ATP-binding protein	5.007564	0.557774	6.72×10^{-13}	3.29×10^{-10}	higher
**HMPREF9571_RS02505**	zinc ABC transporter substrate-10-binding protein	5.997173	0.624211	1.19×10^{-15}	9.7×10^{-13}	higher
**HMPREF9571_RS03560**	N-acetylmuramoyl-L-alanine amidase	3.267527	0.628058	0.000306	0.015593	higher
**HMPREF9571_RS06980**	choice-10-of-anchor M domain-containing protein	4.393113	0.484339	2.46×10^{-12}	8.6×10^{-1}	higher
**HMPREF9571_RS08530**	MFS transporter	2.269512	0.30096	2.46×10^{-5}	0.001589	higher
**HMPREF9571_RS11160**	type B 50S ribosomal protein L31	7.953695	1.192759	5.55×10^{-9}	7.54×10^{-7}	higher
**HMPREF9571_RS11165**	50S ribosomal protein L28	8.822814	1.46438	9.19×10^{-8}	9.37×10^{-6}	higher
**HMPREF9571_RS11170**	50S ribosomal protein L33	8.099995	1.768861	5.97×10^{-5}	0.003566	higher
**HMPREF9571_RS11175**	30S ribosomal protein S14	5.547748	0.639254	1.13×10^{-12}	4.59×10^{-10}	higher
**HMPREF9571_RS11180**	50S ribosomal protein L32	4.044618	0.463306	4.98×10^{-11}	1.02×10^{-8}	higher
**HMPREF9571_RS00045**	hypothetical protein	−3.50685	0.435379	8.52×10^{-9}	1.1×10^{-6}	lower
**HMPREF9571_RS00050**	ATP-binding cassette domain-containing protein	−3.7506	0.4335	2.22×10^{-10}	3.63×10^{-8}	lower
**HMPREF9571_RS00055**	hypothetical protein	−3.85382	0.43286	4.31×10^{-11}	9.6×10^{-9}	lower

**HMPREF9571_RS00060**	hypothetical protein	−5.40846	1.052704	2.82×10^{-5}	0.001768	lower
**HMPREF9571_RS00065**	ABC transporter ATP-binding protein	−3.47092	0.570002	1.46×10^{-5}	0.001115	lower
**HMPREF9571_RS00375**	slipin family protein	−3.94716	0.607007	1.2×10^{-6}	0.000102	lower
**HMPREF9571_RS00380**	hypothetical protein	−4.17448	0.597791	1.09×10^{-7}	1.07×10^{-5}	lower
**HMPREF9571_RS00385**	cbb3-type cytochrome c oxidase subunit I	−4.43107	0.607547	1.63×10^{-8}	1.9×10^{-6}	lower
**HMPREF9571_RS00610**	ribosome-10-associated translation inhibitor RaiA	−2.9401	0.48056	5.41×10^{-5}	0.003311	lower
**HMPREF9571_RS00640**	CAMP factor pore-10-forming toxin 1	−6.27427	0.516058	1.61×10^{-24}	1.97×10^{-21}	lower
**HMPREF9571_RS01270**	sodium-translocating pyrophosphatase	−3.45514	0.472651	2.05×10^{-7}	1.93×10^{-5}	lower
**HMPREF9571_RS03050**	ribonuclease	−3.2985	0.513701	7.66×10^{-6}	0.000605	lower
**HMPREF9571_RS03055**	barstar family protein	−3.48881	0.589157	2.4×10^{-5}	0.001589	lower
**HMPREF9571_RS03340**	hypothetical protein	−2.83681	0.435484	2.47×10^{-5}	0.001589	lower
**HMPREF9571_RS03355**	iron ABC transporter permease	−3.18719	0.573323	0.000136	0.007579	lower
**HMPREF9571_RS04620**	polyphosphate-10--nucleotide phosphotransferase	−2.48969	0.426267	0.000475	0.022778	lower
**HMPREF9571_RS05115**	2-hydroxyacyl-CoA dehydratase	−2.87172	0.526823	0.000381	0.018659	lower
**HMPREF9571_RS06215**	hypothetical protein	−3.97323	0.521385	1.18×10^{-8}	1.44×10^{-6}	lower
**HMPREF9571_RS07645**	pyruvate. phosphate dikinase	−2.71288	0.40257	2.09×10^{-5}	0.001463	lower
**HMPREF9571_RS07670**	response regulator transcription factor	−4.10859	0.462564	1.81×10^{-11}	4.93×10^{-9}	lower
**HMPREF9571_RS08605**	aldehyde dehydrogenase EutE	−2.70486	0.479213	0.000374	0.018659	lower
**HMPREF9571_RS08815**	alpha/beta fold hydrolase	−5.85969	0.658233	1.55×10^{-13}	9.48×10^{-11}	lower
**HMPREF9571_RS08820**	triacylglycerol lipase	−5.50543	0.817507	3.56×10^{-8}	3.97×10^{-6}	lower
**HMPREF9571_RS08925**	MFS transporter	−2.51559	0.391847	0.00011	0.006401	lower
**HMPREF9571_RS09250**	hypothetical protein	−3.88064	0.480108	1.97×10^{-9}	3.02×10^{-7}	lower
**HMPREF9571_RS09255**	hypothetical protein	−3.63155	0.513052	2.91×10^{-7}	2.64×10^{-5}	lower
**HMPREF9571_RS09260**	hypothetical protein	−3.34072	0.397409	3.86×10^{-9}	5.56×10^{-7}	lower
**HMPREF9571_RS09715**	hypothetical protein	−3.21736	0.675411	0.001027	0.047443	lower
**HMPREF9571_RS09950**	RNA polymerase sigma factor	−2.10193	0.337176	0.001083	0.049081	lower
**HMPREF9571_RS09955**	alkaline shock response membrane	−3.18418	0.433568	4.71×10^{-7}	4.12×10^{-5}	lower

	anchor protein AmaP					
**HMPREF9571_RS09960**	hypothetical protein	−2.99677	0.519367	0.000121	0.006874	lower
**HMPREF9571_RS09965**	Asp23/Gls24 family envelope stress response protein	−2.83335	0.499042	0.000239	0.012596	lower
**HMPREF9571_RS09975**	Asp23/Gls24 family envelope stress response protein	−2.97785	0.594976	0.000887	0.041738	lower
**HMPREF9571_RS10620**	DUF2249 domain-containing protein	−2.82504	0.427377	1.95×10^{-5}	0.001405	lower
**HMPREF9571_RS11080**	alpha/beta fold hydrolase	−3.06392	0.456038	6.02×10^{-6}	0.000491	lower
**HMPREF9571_RS11400**	WhiB family transcriptional regulator	−3.9429	0.452384	7.75×10^{-11}	1.46×10^{-08}	lower
**HMPREF9571_RS12255**	hypothetical protein	−4.88263	1.057718	0.000242	0.012596	lower
**HMPREF9571_RS12665**	hypothetical protein	−4.41706	0.490105	3.12×10^{-12}	9.55×10^{-10}	lower
**HMPREF9571_RS12995**	HtaA domain protein	−2.63289	0.430973	0.000151	0.008233	lower
