# Supplementary material for: C-Type Natriuretic Peptide Acts as a Microorganism-Activated Regulator of the Skin Commensals Staphylococcus epidermidis and Cutibacterium acnes in Dual-Species Biofilms
Source: Biology (Basel). 2023 Mar 12;12(3):436. doi: 10.3390/biology12030436 (PMC10045295; doi:10.3390/biology12030436)
Supplement: Supplementary file 1 [file biology-12-00436-s001.zip › Supplementary Table 3.pdf]

Supplementary Table 3. Differential expression of genes in *S. epidermidis* in dual-species biofilms in comparison to *S. epidermidis* monospecies biofilms

Locus tag	Protein description	log2 (expression level ratio)	Standard error of log2	p-value	q-value	Conclusion about expression difference
**B6C95_00220**	RNA polymerase sigma factor SigA	1.767881	0.217896	0.000425	0.035288	higher
**B6C95_00245**	zinc ABC transporter ATP-binding protein	2.66899	0.484208	0.000567	0.041387	higher
**B6C95_01415**	hypothetical protein	5.811647	0.785455	9.02×10^{-10}	1.81×10^{-07}	higher
**B6C95_01420**	thermonuclease	7.168621	0.618745	2.07×10^{-23}	9.98×10^{-21}	higher
**B6C95_01430**	DNA-binding response regulator	7.146496	0.55497	1.65×10^{-28}	9.95×10^{-26}	higher
**B6C95_01435**	sensor histidine kinase	7.233016	0.442518	4.67×10^{-45}	5.63×10^{-42}	higher
**B6C95_01440**	ABC transporter permease	7.937752	0.429995	1.46×10^{-58}	3.51×10^{-55}	higher
**B6C95_01445**	antibiotic ABC transporter ATP-binding protein	8.43166	0.624973	1.32×10^{-32}	1.06×10^{-29}	higher
**B6C95_02450**	lysostaphin resistance protein A	2.311044	0.331911	7.82×10^{-05}	0.00808	higher
**B6C95_03495**	CHAP domain-containing protein	4.259572	0.73121	8.28×10^{-06}	0.001108	higher
**B6C95_05745**	ATP-dependent Clp protease ATP-binding subunit ClpX	1.653574	0.186898	0.000471	0.036871	higher
**B6C95_06070**	signal recognition particle sRNA large type	5.115595	0.67031	8.26×10^{-10}	1.81×10^{-07}	higher
**B6C95_09440**	transglycosylase IsaA	2.757612	0.502943	0.000475	0.036871	higher
**B6C95_09980**	transglycosylase SceD	6.024359	0.747224	1.77×10^{-11}	4.73×10^{-09}	higher
**B6C95_10420**	hypothetical protein	2.857432	0.402271	3.89×10^{-06}	0.000585	higher
**B6C95_11955**	CHAP domain-containing protein	4.760818	0.953838	8.05×10^{-05}	0.00808	higher
**B6C95_12180**	CHAP domain-containing protein	4.289043	0.736542	7.99×10^{-06}	0.001108	higher
**B6C95_02215**	succinate dehydrogenase	−2.88487	0.440113	1.85×10^{-05}	0.002117	lower
**B6C95_02220**	hypothetical protein	−4.28445	0.568847	7.75×10^{-09}	1.43×10^{-06}	lower
**B6C95_02685**	PTS maltose transporter subunit IIBC	−3.23046	0.423424	1.38×10^{-07}	2.38×10^{-05}	lower
**B6C95_03235**	hypothetical protein	−3.70183	0.612677	1.03×10^{-05}	0.001264	lower
**B6C95_04760**	hypothetical protein	−7.34486	1.250863	3.93×10^{-07}	6.31×10^{-05}	lower
**B6C95_04810**	hypothetical protein	−2.9864	0.499728	7.04×10^{-05}	0.007704	lower

**B6C95_04815**	TIGR00268 family protein	−2.82089	0.486534	0.000182	0.016869	lower
**B6C95_06910**	hypothetical protein	−3.42944	0.661225	0.000239	0.021283	lower
**B6C95_07005**	glutamate dehydrogenase	−2.44688	0.410052	0.000418	0.035288	lower
**B6C95_08375**	lipase	−4.93599	0.551422	9.48×10^{-13}	2.85×10^{-10}	lower
**B6C95_08380**	hypothetical protein	−5.99567	0.69703	7.66×10^{-13}	2.64×10^{-10}	lower
**B6C95_10465**	mannose-6-phosphate isomerase, class I	−5.48209	0.719807	4.76×10^{-10}	1.15×10^{-07}	lower
**B6C95_10470**	PTS mannose transporter subunit IIABC	−6.8882	0.628282	7.12×10^{-21}	2.86×10^{-18}	lower
**B6C95_10920**	tRNA-Asp	−4.05737	0.815584	0.000178	0.016869	lower
**B6C95_10960**	tRNA-Gly	−3.55163	0.579044	1.05×10^{-05}	0.001264	lower
**B6C95_12215**	tRNA-Ala	−2.97504	0.5685	0.000513	0.038566	lower
