# Supplementary material for: C-Type Natriuretic Peptide Acts as a Microorganism-Activated Regulator of the Skin Commensals Staphylococcus epidermidis and Cutibacterium acnes in Dual-Species Biofilms
Source: Biology (Basel). 2023 Mar 12;12(3):436. doi: 10.3390/biology12030436 (PMC10045295; doi:10.3390/biology12030436)
Supplement: Supplementary file 1 [file biology-12-00436-s001.zip › Supplementary Table 4.pdf]

Supplementary table 4. The contamination analysis in the RNA-seq samples

Species	Sample	
	***Cutibacterium acnes*, CNP, replicate 1**	
**Best matching species**	**Number of reads with this best matching species**	**Family of the best matching species**
Cutibacterium acnes	964	*Propionibacteriaceae*
**no matches**	10	
**uncultured bacterium**	8	information on family is absent in NCBI Taxonomy
Propionibacterium freudenreichii	5	*Propionibacteriaceae*
Burmannia oblonga	2	*Burmanniaceae*
**uncultured organism**	1	information on family is absent in NCBI Taxonomy
Bifidobacterium animalis	1	*Bifidobacteriaceae*
***Actinomadura sp.* NAK00032**	1	*Thermomonosporaceae*
Acidipropionibacterium jensenii	1	*Propionibacteriaceae*
***Sanguibacter sp.* HDW7**	1	*Sanguibacteraceae*
***Arcanobacterium sp.* 2701**	1	*Actinomycetaceae*
Cutibacterium avidum	1	*Propionibacteriaceae*
***Burmannia sp.* KH-2014**	1	*Burmanniaceae*
Enterococcus avium	1	*Enterococcaceae*
Mycolicibacterium sp.	1	*Mycobacteriaceae*
Burmannia itoana	1	*Burmanniaceae*
	***Cutibacterium acnes*, CNP, replicate 2**	
**Best matching species**	**Number of reads with this best matching species**	**Family of the best matching species**
Cutibacterium acnes	969	*Propionibacteriaceae*
Propionibacterium freudenreichii	14	*Propionibacteriaceae*
**no matches**	7	
**uncultured bacterium**	2	information on family is absent in NCBI Taxonomy
***Sanguibacter sp.* HDW7**	1	*Sanguibacteraceae*
***Arcanobacterium sp.* 2701**	1	*Actinomycetaceae*
Enterococcus avium	1	*Enterococcaceae*
Homo sapiens	1	*Hominidae*
Kocuria sp.	1	*Micrococcaceae*
Bifidobacterium animalis	1	*Bifidobacteriaceae*
***Actinomyces sp.* oral taxon 848**	1	*Actinomycetaceae*
***Nocardioides sp.* JQ2195**	1	*Nocardioidaceae*

Cutibacterium acnes, CNP, replicate 3		
**Best matching species**	**Number of reads with this best matching species**	**Family of the best matching species**
Cutibacterium acnes	964	*Propionibacteriaceae*
**no matches**	10	
Propionibacterium freudenreichii	4	*Propionibacteriaceae*
**uncultured bacterium**	4	information on family is absent in NCBI Taxonomy
Enterococcus avium	4	*Enterococcaceae*
Cutibacterium modestum	2	*Propionibacteriaceae*
***Brevibacterium* sp. 'Marine'**	1	*Brevibacteriaceae*
Brevibacterium sediminis	1	*Brevibacteriaceae*
***Nocardioides* sp. JQ2195**	1	*Nocardioidaceae*
Deinococcus wulumuqiensis	1	*Deinococcaceae*
**eukaryotic synthetic construct**	1	information on family is absent in NCBI Taxonomy
Staphylococcus epidermidis	1	*Staphylococcaceae*
Brevilactibacter coleopterorum	1	*Propionibacteriaceae*
Streptococcus salivarius	1	*Streptococcaceae*
***Burmannia* sp. KH-2014**	1	*Burmanniaceae*
Cutibacterium avidum	1	*Propionibacteriaceae*
Nosocomiicoccus ampullae	1	*Staphylococcaceae*
***Arcanobacterium* sp. 2701**	1	*Actinomycetaceae*

Cutibacterium acnes, control, replicate 1		
**Best matching species**	**Number of reads with this best matching species**	**Family of the best matching species**
Cutibacterium acnes	982	*Propionibacteriaceae*
Propionibacterium freudenreichii	6	*Propionibacteriaceae*
Cutibacterium avidum	3	*Propionibacteriaceae*
Enterococcus avium	2	*Enterococcaceae*
Cutibacterium modestum	1	*Propionibacteriaceae*
***Pseudomonas* sp.**	*1*	*Pseudomonadaceae*
***Nocardioides* sp. JQ2195**	1	*Nocardioidaceae*
**no matches**	1	
Pseudomonas psychrotolerans	1	*Pseudomonadaceae*
Pseudomonas oryzihabitans	1	*Pseudomonadaceae*
**uncultured bacterium**	1	information on family is absent in NCBI Taxonomy

Cutibacterium acnes, control, replicate 2		
**Best matching species**	**Number of reads with this best matching species**	**Family of the best matching species**
Cutibacterium acnes	984	*Propionibacteriaceae*
Cutibacterium modestum	3	*Propionibacteriaceae*
Acidipropionibacterium jensenii	2	*Propionibacteriaceae*
**uncultured bacterium**	2	information on family is absent in NCBI Taxonomy
Cutibacterium avidum	2	*Propionibacteriaceae*
**no matches**	2	
Bifidobacterium animalis	1	*Bifidobacteriaceae*
Pseudomonas oryzihabitans	1	*Pseudomonadaceae*
Brevilactibacter coleopterorum	1	*Propionibacteriaceae*
Propionibacterium freudenreichii	1	*Propionibacteriaceae*
**uncultured Tessaracoccus sp.**	1	*Propionibacteriaceae*

Cutibacterium acnes, control, replicate 3		
**Best matching species**	**Number of reads with this best matching species**	**Family of the best matching species**
Cutibacterium acnes	972	*Propionibacteriaceae*
Enterococcus avium	6	*Enterococcaceae*
Cutibacterium modestum	5	*Propionibacteriaceae*
Propionibacterium freudenreichii	4	*Propionibacteriaceae*
**uncultured bacterium**	4	information on family is absent in NCBI Taxonomy
**no matches**	4	
Pseudomonas oryzihabitans	2	*Pseudomonadaceae*
***Nocardioides* sp.**	1	*Nocardioidaceae*
Homo sapiens	1	*Hominidae*
Acidipropionibacterium jensenii	1	*Propionibacteriaceae*

Cutibacterium acnes, control, replicate 4		
**Best matching species**	**Number of reads with this best matching species**	**Family of the best matching species**
Cutibacterium acnes	979	*Propionibacteriaceae*
Propionibacterium freudenreichii	4	*Propionibacteriaceae*
**no matches**	3	
Enterococcus avium	2	*Enterococcaceae*
Burmannia oblonga	1	*Burmanniaceae*

Cutibacterium modestum	1	*Propionibacteriaceae*
***Nocardioides* sp. MC1495**	1	*Nocardioidaceae*
***Kocuria* sp.**	1	*Micrococcaceae*
Corynebacterium pseudotuberculosis	1	*Corynebacteriaceae*
Propionibacteriaceae bacterium	1	*Propionibacteriaceae*
Acidipropionibacterium jensenii	1	*Propionibacteriaceae*
Cutibacterium avidum	1	*Propionibacteriaceae*
***Burmannia* sp. KH-2014**	1	*Burmanniaceae*
Sphingomonas hengshuiensis	1	*Sphingomonadaceae*
**uncultured bacterium**	1	information on family is absent in NCBI Taxonomy
**eukaryotic synthetic construct**	1	information on family is absent in NCBI Taxonomy
***Cutibacterium acnes*, control, replicate 5**		
**Best matching species**	**Number of reads with this best matching species**	**Family of the best matching species**
Cutibacterium acnes	986	*Propionibacteriaceae*
**no matches**	4	
Propionibacterium freudenreichii	2	*Propionibacteriaceae*
Clostridium thermarum	1	*Clostridiaceae*
***Microbacterium* sp. ALBL_076**	1	*Microbacteriaceae*
Staphylococcus epidermidis	1	*Staphylococcaceae*
Enterococcus avium	1	*Enterococcaceae*
Cutibacterium avidum	1	*Propionibacteriaceae*
Acidipropionibacterium acidipropionici	1	*Propionibacteriaceae*
***Kocuria* sp.**	1	*Micrococcaceae*
***Amycolatopsis* sp. Hca4**	1	*Pseudonocardiaceae*
***Staphylococcus epidermidis*, CNP, replicate 1**		
**Best matching species**	**Number of reads with this best matching species**	**Family of the best matching species**
Staphylococcus epidermidis	938	*Staphylococcaceae*
Staphylococcus cohnii	29	*Staphylococcaceae*
Staphylococcus aureus	11	*Staphylococcaceae*
Staphylococcus hominis	10	*Staphylococcaceae*
**no matches**	3	
**eukaryotic synthetic construct**	2	information on family is absent in NCBI Taxonomy
Homo sapiens	2	*Hominidae*
Felis catus	1	*Felidae*

***Burkholderiales* bacterium**	1	information on family is absent in NCBI Taxonomy
Micrococcus luteus	1	*Micrococcaceae*
Cutibacterium acnes	1	*Propionibacteriaceae*
Staphylococcus saprophyticus	1	*Staphylococcaceae*
	***Staphylococcus epidermidis*, CNP, replicate 2**	
**Best matching species**	**Number of reads with this best matching species**	**Family of the best matching species**
Staphylococcus epidermidis	931	*Staphylococcaceae*
Staphylococcus cohnii	35	*Staphylococcaceae*
Staphylococcus aureus	17	*Staphylococcaceae*
Staphylococcus hominis	12	*Staphylococcaceae*
Homo sapiens	2	*Hominidae*
Staphylococcus saprophyticus	1	*Staphylococcaceae*
Candidatus Rhabdochlamydia porcellionis	1	*Rhabdochlamydiaceae*
***Schizophyllum* sp.**	1	*Schizophyllaceae*
**no matches**	0	
	***Staphylococcus epidermidis*, CNP, replicate 3**	
**Best matching species**	**Number of reads with this best matching species**	**Family of the best matching species**
Staphylococcus epidermidis	943	*Staphylococcaceae*
Staphylococcus cohnii	20	*Staphylococcaceae*
Staphylococcus aureus	12	*Staphylococcaceae*
Staphylococcus hominis	9	*Staphylococcaceae*
**no matches**	8	
Cutibacterium acnes	2	*Propionibacteriaceae*
Homo sapiens	2	*Hominidae*
Bacillus anthracis	1	*Bacillaceae*
**uncultured bacterium**	1	information on family is absent in NCBI Taxonomy
Sulfitobacter donghicola	1	*Roseobacteraceae*
Staphylococcus saprophyticus	1	*Staphylococcaceae*
	***Staphylococcus epidermidis*, control, replicate 1**	
**Best matching species**	**Number of reads with this best matching species**	**Family of the best matching species**

**Staphylococcus epidermidis**	956	Staphylococcaceae
**Staphylococcus hominis**	17	Staphylococcaceae
**Staphylococcus cohnii**	11	Staphylococcaceae
**Staphylococcus aureus**	5	Staphylococcaceae
**Homo sapiens**	4	Hominidae
**no matches**	2	
**Staphylococcus haemolyticus**	1	Staphylococcaceae
**Cutibacterium acnes**	1	Propionibacteriaceae
**Micrococcus sp.**	1	Micrococcaceae
**Corynebacterium tuberculostearicum**	1	Corynebacteriaceae
**Lolium perenne**	1	Poaceae
	***Staphylococcus epidermidis*, control, replicate 2**	
**Best matching species**	**Number of reads with this best matching species**	**Family of the best matching species**
Staphylococcus epidermidis	872	*Staphylococcaceae*
Homo sapiens	40	*Hominidae*
**no matches**	19	
**eukaryotic synthetic construct**	16	information on family is absent in NCBI Taxonomy
Staphylococcus hominis	13	*Staphylococcaceae*
Staphylococcus cohnii	7	*Staphylococcaceae*
Cutibacterium acnes	5	*Propionibacteriaceae*
Staphylococcus aureus	4	*Staphylococcaceae*
**uncultured bacterium**	2	information on family is absent in NCBI Taxonomy
Pan troglodytes	2	*Hominidae*
Naegleria fowleri	1	*Vahlkampfiidae*
Macrococcus caseolyticus	1	*Staphylococcaceae*
Corynebacterium tuberculostearicum	1	*Corynebacteriaceae*
Sus scrofa	1	*Suidae*
Corynebacterium imitans	1	*Corynebacteriaceae*
Kocuria rosea	1	*Micrococcaceae*
Novosphingobium resinovorum	1	*Sphingomonadaceae*
**Severe acute respiratory syndrome-related coronavirus**	1	*Coronaviridae*
Streptococcus oralis	1	*Streptococcaceae*
Pinus yunnanensis	1	*Pinaceae*
Vagococcus fluvialis	1	*Enterococcaceae*
***Corynebacterium* sp. 4H37-19**	1	*Corynebacteriaceae*
Staphylococcus capitis	1	*Staphylococcaceae*
Moraxella osloensis	1	*Moraxellaceae*
Corynebacterium ureicelerivorans	1	*Corynebacteriaceae*

Alternaria alternata	1	*Pleosporaceae*
Finegoldia magna	1	*Peptoniphilaceae*
Pan paniscus	1	*Hominidae*
Staphylococcus saprophyticus	1	*Staphylococcaceae*
Moellerella wisconsensis	1	*Morganellaceae*
	***Staphylococcus epidermidis*, control, replicate 3**	
**Best matching species**	**Number of reads with this best matching species**	**Family of the best matching species**
Staphylococcus epidermidis	918	*Staphylococcaceae*
Staphylococcus hominis	19	*Staphylococcaceae*
Staphylococcus cohnii	17	*Staphylococcaceae*
Homo sapiens	12	*Hominidae*
Staphylococcus aureus	10	*Staphylococcaceae*
**eukaryotic synthetic construct**	6	information on family is absent in NCBI Taxonomy
Pan troglodytes	2	*Hominidae*
Fabriciana adippe	1	*Nymphalidae*
Oncorhynchus mykiss	1	*Salmonidae*
Micrococcus luteus	1	*Micrococcaceae*
Acinetobacter junii	1	*Moraxellaceae*
Malus sylvestris	1	*Rosaceae*
Coregonus sp. 'balchen'	1	*Salmonidae*
Microbacterium sp. No. 7	1	*Microbacteriaceae*
Sphingomonas sp. HMP9	1	*Sphingomonadaceae*
Festuca sicula	1	*Poaceae*
Psychrobacter sp. WY6	1	*Moraxellaceae*
Moraxella osloensis	1	*Moraxellaceae*
**no matches**	1	
Isosphaera pallida	1	*Isosphaeraceae*
Brevundimonas phage vB_BsubS-Delta	1	*Siphoviridae*
Neosetophoma aseptata	1	*Phaeosphaeriaceae*
Shigella sonnei	1	*Enterobacteriaceae*
	**Dual-species, CNP, replicate 1**	
**Best matching species**	**Number of reads with this best matching species**	**Family of the best matching species**
Staphylococcus epidermidis	723	*Staphylococcaceae*
Cutibacterium acnes	218	*Propionibacteriaceae*
Staphylococcus aureus	14	*Staphylococcaceae*

Staphylococcus hominis	11	*Staphylococcaceae*
Staphylococcus cohnii	8	*Staphylococcaceae*
**no matches**	5	
Staphylococcus saprophyticus	3	*Staphylococcaceae*
**eukaryotic synthetic construct**	3	information on family is absent in NCBI Taxonomy
***Acinetobacter* sp. NEB 394**	2	*Moraxellaceae*
Homo sapiens	2	*Hominidae*
Propionibacterium freudenreichii	2	*Propionibacteriaceae*
Acinetobacter junii	1	*Moraxellaceae*
Secale cereale	1	*Poaceae*
Cutibacterium avidum	1	*Propionibacteriaceae*
Streptococcus equi	1	*Streptococcaceae*
Enterococcus avium	1	*Enterococcaceae*
Triticum aestivum	1	*Poaceae*
**uncultured bacterium**	1	information on family is absent in NCBI Taxonomy
**bacterium**	1	information on family is absent in NCBI Taxonomy
Cortinarius mucifluus	1	*Cortinariaceae*
**Dual-species, CNP, replicate 2**		
**Best matching species**	**Number of reads with this best matching species**	**Family of the best matching species**
Cutibacterium acnes	894	*Propionibacteriaceae*
Staphylococcus epidermidis	84	*Staphylococcaceae*
Propionibacterium freudenreichii	4	*Propionibacteriaceae*
Homo sapiens	3	*Hominidae*
**uncultured bacterium**	3	information on family is absent in NCBI Taxonomy
Propionibacteriaceae bacterium	2	*Propionibacteriaceae*
**no matches**	2	
Acidipropionibacterium jensenii	1	*Propionibacteriaceae*
Cutibacterium modestum	1	*Propionibacteriaceae*
**eukaryotic synthetic construct**	1	information on family is absent in NCBI Taxonomy
Staphylococcus aureus	1	*Staphylococcaceae*
Capsicum annuum	1	*Solanaceae*
Sulfitobacter donghicola	1	*Roseobacteraceae*
Staphylococcus cohnii	1	*Staphylococcaceae*
Sphingomonas sinipercae	1	*Sphingomonadaceae*

Dual-species, CNP, replicate 3

Best matching species	Number of reads with this best matching species	Family of the best matching species
Staphylococcus epidermidis	631	*Staphylococcaceae*
Cutibacterium acnes	318	*Propionibacteriaceae*
Staphylococcus cohnii	15	*Staphylococcaceae*
Staphylococcus aureus	13	*Staphylococcaceae*
Staphylococcus hominis	7	*Staphylococcaceae*
Homo sapiens	4	*Hominidae*
Propionibacterium freudenreichii	3	*Propionibacteriaceae*
Cutibacterium avidum	2	*Propionibacteriaceae*
Solidago gigantea	1	*Asteraceae*
**uncultured bacterium**	1	information on family is absent in NCBI Taxonomy
Delftia acidovorans	1	*Comamonadaceae*
Abies nordmanniana	1	*Pinaceae*
**eukaryotic synthetic construct**	1	information on family is absent in NCBI Taxonomy
***Paratricoma* sp. 1L13G15**	1	*Desmoscolecidae*
Lacipirellula parvula	1	*Lacipirellulaceae*
**no matches**	0	

Dual-species, control, replicate 1

Best matching species	Number of reads with this best matching species	Family of the best matching species
Staphylococcus epidermidis	829	*Staphylococcaceae*
Cutibacterium acnes	133	*Propionibacteriaceae*
Staphylococcus hominis	10	*Staphylococcaceae*
Staphylococcus cohnii	9	*Staphylococcaceae*
Staphylococcus aureus	7	*Staphylococcaceae*
**no matches**	6	
Enterococcus avium	2	*Enterococcaceae*
Homo sapiens	2	*Hominidae*
Staphylococcus saprophyticus	1	*Staphylococcaceae*
Micrococcus luteus	1	*Micrococcaceae*

Dual-species, control, replicate 2

Best matching species	Number of reads with this best matching species	Family of the best matching species

Staphylococcus epidermidis	755	*Staphylococcaceae*
Cutibacterium acnes	201	*Propionibacteriaceae*
Staphylococcus aureus	12	*Staphylococcaceae*
Staphylococcus hominis	11	*Staphylococcaceae*
Staphylococcus cohnii	6	*Staphylococcaceae*
Staphylococcus saprophyticus	3	*Staphylococcaceae*
**no matches**	3	
Staphylococcus pseudintermedius	2	*Staphylococcaceae*
***Massilia* sp. WG5**	1	*Oxalobacteraceae*
Priestia megaterium	1	*Bacillaceae*
Homo sapiens	1	*Hominidae*
Acidipropionibacterium jensenii	1	*Propionibacteriaceae*
Comamonas testosteroni	1	*Comamonadaceae*
Propionibacterium freudenreichii	1	*Propionibacteriaceae*
**eukaryotic synthetic construct**	1	information on family is absent in NCBI Taxonomy
**Dual-species, control, replicate 3**		
**Best matching species**	**Number of reads with this best matching species**	**Family of the best matching species**
Cutibacterium acnes	917	*Propionibacteriaceae*
Staphylococcus epidermidis	65	*Staphylococcaceae*
Propionibacterium freudenreichii	4	*Propionibacteriaceae*
**no matches**	3	
**uncultured bacterium**	3	information on family is absent in NCBI Taxonomy
***Microbacterium* sp. Marseille-Q2854**	2	*Microbacteriaceae*
Cutibacterium modestum	1	*Propionibacteriaceae*
***Kocuria* sp.**	1	*Micrococcaceae*
Homo sapiens	1	*Hominidae*
Acidipropionibacterium acidipropionici	1	*Propionibacteriaceae*
Staphylococcus hominis	1	*Staphylococcaceae*
Glutamicibacter mishrai	1	*Micrococcaceae*
