# Supplementary material for: C-Type Natriuretic Peptide Acts as a Microorganism-Activated Regulator of the Skin Commensals Staphylococcus epidermidis and Cutibacterium acnes in Dual-Species Biofilms
Source: Biology (Basel). 2023 Mar 12;12(3):436. doi: 10.3390/biology12030436 (PMC10045295; doi:10.3390/biology12030436)
Supplement: Supplementary file 1 [file biology-12-00436-s001.zip › Supplementary table 5.pdf]

Supplementary table. Primers used for qPCR

Gene	#	Primer	Sequence
***Cutibacterium acnes* HMPREF9571_RS01535 TIGR03773 family transporter-associated surface protein**	1	Forward primer	5'-CAGACAGAGAGAGCACCGTC-3'
	2	Reverse primer	5'-ATCTGGTGCATGGTGTGGAG-3'
***Cutibacterium acnes* HMPREF9571_RS02495 metal ABC transporter permease**	3	Forward primer	5'-ACAGATCACGCTGTTGCAGA-3'
	4	Reverse primer	5'-GTAGGGCGTCCGAGAATGAG-3'
***Cutibacterium acnes* HMPREF9571_RS06980 choice-of-anchor M domain-containing protein**	5	Forward primer	5'-CACGAGCTACGAAGGAGCAG-3'
	6	Reverse primer	5'-AGTTACGAAGTCCTCCCCGA-3'
***Cutibacterium acnes* HMPREF9571_RS11175 30S ribosomal protein S14**	7	Forward primer	5'-TACCCTCTGCTGCCATTTCG-3'
	8	Reverse primer	5'-AAAGATCAGCGAGGACTGCC-3'
***Cutibacterium acnes* 16S rRNA**	9	Forward primer	5'-GTAAACCGCTTTCGCCTGTG-3'
	10	Reverse primer	5'-CAACCACCTACGAGCCCTTT-3'
***Cutibacterium acnes* HMPREF9571_RS11165 50S ribosomal protein L28**	11	Forward primer	ACGGCAATACTTACCCCGTC
	12	Reverse primer	AACTTCTCAACTCGTCCGGC
***Staphylococcus epidermidis* B6C95_06200 hypothetical protein**	13		5'- TGAAGAAATGCCCGTCCCTC-3"
	14		5'- TCGAGTGGGTTCAATGCGAA-3'
***Staphylococcus epidermidis* NADH-quinone oxidoreductase subunit L**	15	Forward primer	5'-ACAGATGGAGGCGTAGGAATG-3'
	16	Reverse primer	5'-AGTACAATGTGCGCTTGGGG-3'
***Staphylococcus epidermidis* 16S rRNA**	17	Forward primer	5'-CGAAAGCGTGGGGATCAAAC-3'
	18	Reverse primer	5'-TCAACCTTGCGGTCGTACTC-3'
