# Supplementary figures and images for: C-Type Natriuretic Peptide Acts as a Microorganism-Activated Regulator of the Skin Commensals Staphylococcus epidermidis and Cutibacterium acnes in Dual-Species Biofilms
Source: Biology (Basel). 2023 Mar 12;12(3):436. doi: 10.3390/biology12030436 (PMC10045295; doi:10.3390/biology12030436)

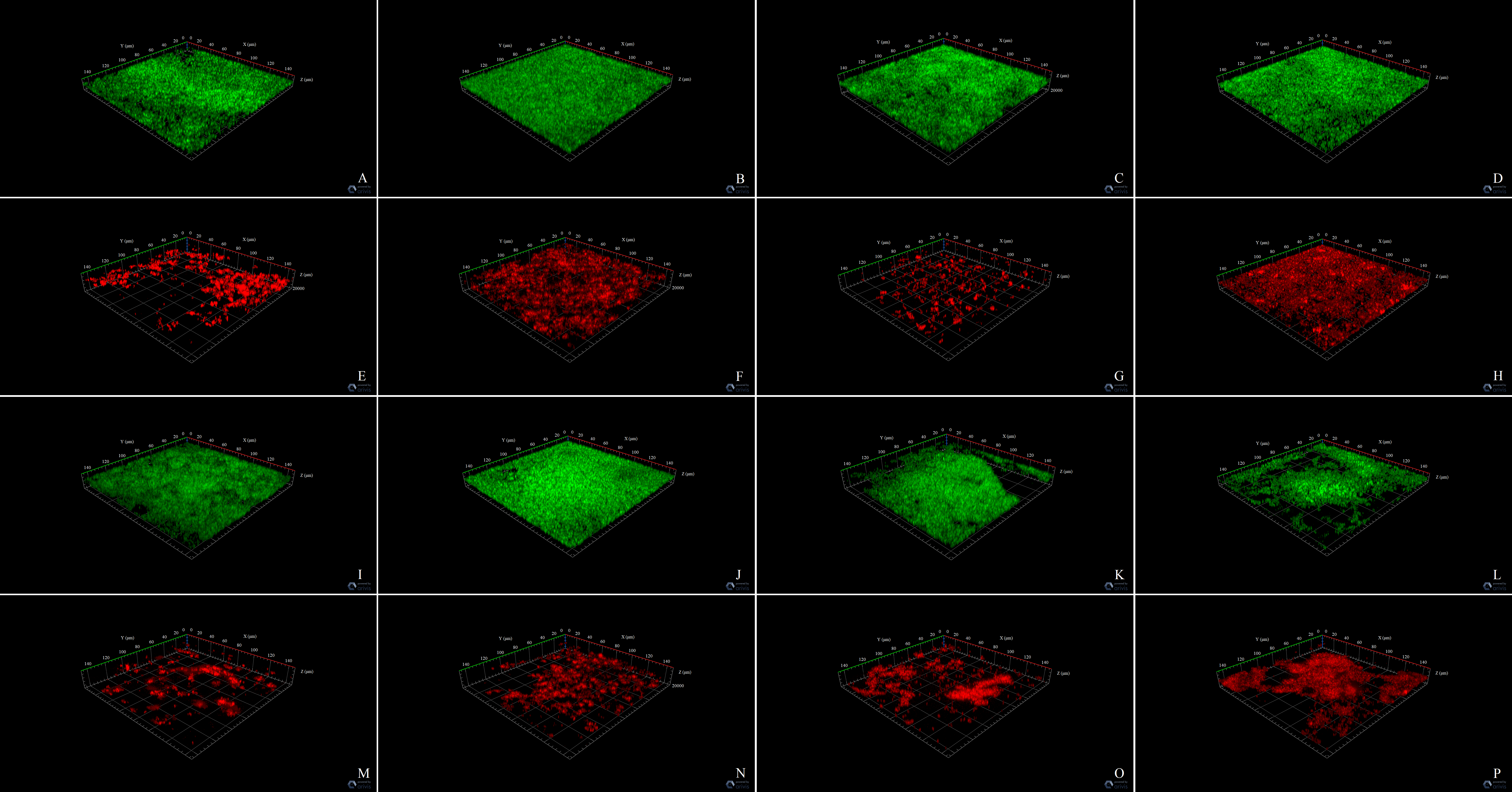

Supplement: Supplementary file 1 [file biology-12-00436-s001.zip › Supplementary figure 1.tif]

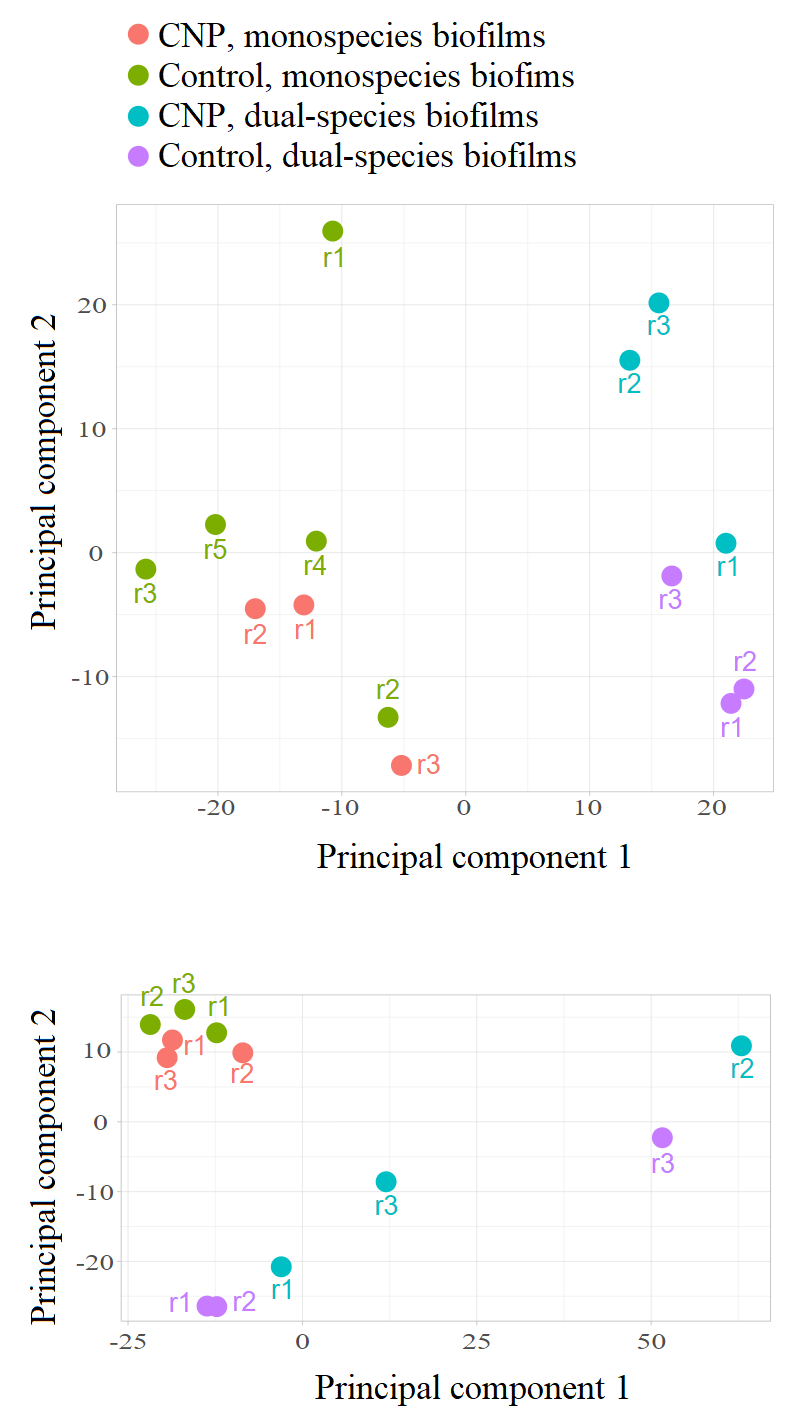

Supplement: Supplementary file 1 [file biology-12-00436-s001.zip › Supplementary figure 2.tif]
